# Direct Observation of the ππ* to nπ* Transition in 2-Thiouracil via Time-Resolved NEXAFS Spectroscopy

**DOI:** 10.1021/acs.jpclett.5c00544

**Published:** 2025-04-15

**Authors:** Fabiano Lever, David Picconi, Dennis Mayer, Skirmantas Ališauskas, Francesca Calegari, Stefan Düsterer, Raimund Feifel, Marion Kuhlmann, Tommaso Mazza, Jan Metje, Matthew S. Robinson, Richard J. Squibb, Andrea Trabattoni, Matthew Ware, Peter Saalfrank, Thomas J. A. Wolf, Markus Gühr

**Affiliations:** † Deutsches Elektronen-Synchrotron DESY, Hamburg, 22607, Germany; ‡ Heinrich-Heine University, Düsseldorf, 40225, Germany; § The Hamburg Centre for Ultrafast Imaging, Hamburg, 20148, Germany; ∥ University of Gothenburg, Gothenburg, 405 30, Sweden; ⊥ European XFEL, Schenefeld, 22869, Germany; # University of Potsdam, Potsdam, 14469, Germany; 7 Leibniz University Hannover, Hannover, 30060, Germany; 8 Stanford PULSE Institute, SLAC National Accelerator Laboratory, Stanford, California 94305, United States

## Abstract

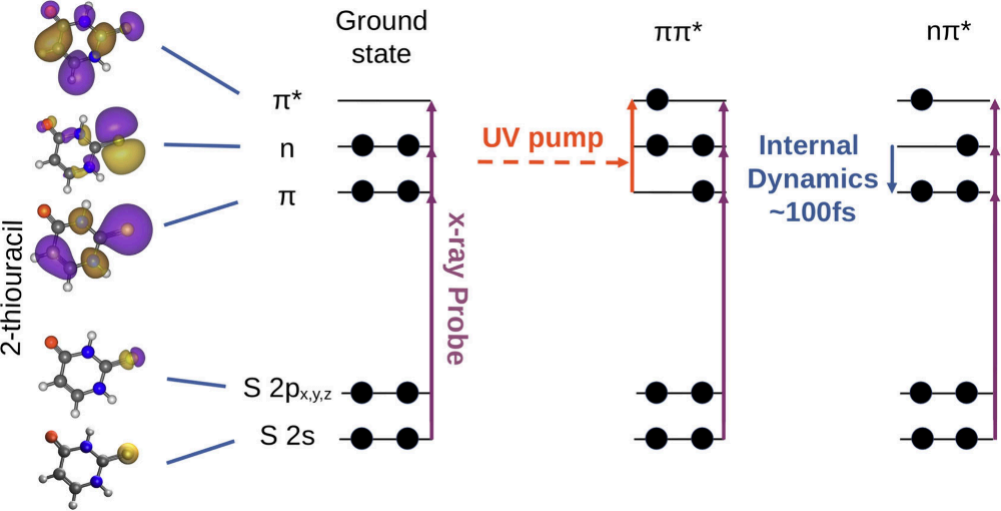

The photophysics of nucleobases has been the subject of both theoretical and experimental studies over the past decades due to the challenges posed by resolving the steps of their radiationless relaxation dynamics, which cannot be described in the framework of the Born–Oppenheimer approximation (BOA). In this context, the ultrafast dynamics of 2-thiouracil has been investigated with a time-resolved NEXAFS study at the Free Electron Laser FLASH. Near Edge X-ray Absorption Fine Structure spectroscopy (NEXAFS) can be used to observe electronic transitions in ultrafast molecular relaxation. We performed time-resolved UV-pump/X-ray probe absorption measurements at the sulfur 2*s* (L1) and 2*p* (L2/3) edges. We are able to identify absorption features corresponding to the S2 (ππ*) and S1 (nπ*) electronic states. We observe a delay of 102 ± 11 fs in the population of the nπ* state with respect to the initial optical excitation and interpret the delay as the time scale for the S2 → S1 internal conversion. We furthermore identify oscillations in the absorption signal that match a similar observation in our previous X-ray photoelectron spectroscopy study on the same molecule.

Modeling the conversion of energy in molecules from absorbed light into different energetic forms is fundamentally important in understanding the physics of many molecular systems. In molecules, energy from visible and ultraviolet light is initially absorbed in the form of electronic excitation, and conversion into new chemical bonds, change in the geometry, or heat occurs via the coupling of the electronic and nuclear degrees of freedom. In such highly coupled systems, electronic and nuclear dynamics proceed on similar time scales, and the Born–Oppenheimer approximation (BOA) cannot be applied.^1,2^ These factors make the study of these processes challenging both theoretically and experimentally.

The efficient dissipation of excess energy as heat is of fundamental importance in the photophysics of nucleobases, as it limits the rate of photoinduced lesions in DNA caused by the exposure to ultraviolet (UV) light from the sun.^3^ Canonical nucleobases show remarkable stability against photoinduced damage, thanks to ultrafast relaxation to the electronic ground state via internal conversion and Intersystem Crossing (ISC).^4−7^

Thionucleobases are obtained from their canonical counterparts by the replacement of one or more oxygen atoms with sulfur. When compared to canonical nucleobases, photoexcitation leads to long-lived excited triplet states, leading to cross-linking reactions^8,9^ and the creation of reactive singlet oxygen.^10,11^ This is important in the context of the medical use of these molecules as immunosuppressants.^12^ In addition, their absorption spectrum is shifted from the UVC region into the UVA^13−15^ - which is more abundant in the sun’s spectrum - further compounding the effects of their reactive excited state. Canonical pyrimidine nucleobases in the gas phase show that the microscopic origin of the changed relaxation dynamics lies in the changed potential energy landscape and increased spin–orbit coupling due to the heavy atom substitution. The conical intersection to the ground state is less accessible in thionucleobases compared to the canonical counterpart due to the excited state redshift. The added increased spin–orbit coupling renders the intersystem crossing more efficient in competition to the ground state relaxation.^15^

2-Thiouracil (2-TU) is the most-studied thionucleobase and serves as a benchmark for the study of the photodynamical pathways that follow UV excitation in this class of molecules.^11,14,16−23^ The absorption of a UV photon excites the molecule to the S_2_ state,^24^ with ^1^ππ* electronic character, which is found to relax to a triplet state on a picosecond time scale.^15^ The S_1_^1^nπ* state is thought to act as intermediate in the relaxation mechanism.^20,25−27^ A competing singlet-ground state relaxation pathway has been proposed^17,23,25,28^ for a ∼30% fraction of the excited population, limiting the effective ISC yield. The precise ^1^ππ* to ^1^nπ* transition time constant is the subject of research, with theoretical studies reporting values ranging from 60 fs^29^ to 250 fs,^30^ depending on the method used. Valence photoelectron studies report experimental time constants below ∼80 fs,^23^ attributing it to electronic relaxation. While in the case of canonical pyrimidine nucleobases, the ^1^nπ* to triplet state relaxation takes several picoseconds (see, e.g., refs (31 and 32)), the increased spin–orbit coupling upon thionation shortens this process to the few 100 fs range.^15^

Our previous work on thymine^31^ has shown that for a heteroaromatic molecule X-ray absorption allows a direct probing of the ^1^nπ* population. The lone pair *n* orbital, which is half filled in the ^1^nπ* state, is essentially a valence *p* orbital of a heteroatom. Due to its strong localization, its occupation can be potentially probed by a transition from a core s orbital into the half-filled *p* valence orbital. In the case of 2-TU, the *n*-orbital is a sulfur 3*p* valence orbital. In this work, we present time-resolved 2*s → n* absorption as a pre-edge feature of the sulfur L_1_ edge. In addition, we show the time-resolved L_2,3_ edges, where a bleach can be identified in 2*p* → π* transitions. We interpret the latter as the ground-state bleach and ππ* excitation feature, while the former directly shows the onset of the nπ* population. We can identify a delay of 102 ± 11 fs in the rise time of features attributed to the ^1^ππ* to ^1^nπ* transition. We furthermore observe coherent oscillations that we attribute to an oscillation of the S_1_ (^1^nπ*) population, as we previously reported in our X-ray photoelectron (XPS) study.^25^

The data was recorded in a UV-pump/X-ray probe setup, allowing for the collection of photo- and Auger-electron spectra in a Magnetic Bottle time-of-flight Electron Spectrometer (MBES).^33^ The experiment was performed at the FL24 beamline of the free electron laser FLASH2^34,35^ (Hamburg, DE), using the purpose-built URSA-PQ apparatus.^36^ The sample was purchased from Sigma-Aldrich with purity ≥ 99%. A capillary oven was used to heat the sample to 150 °C and evaporate it, delivering it to the interaction region in the gas phase. Details on the oven construction can be found in ref (37).

The sample was investigated in two different regimes. Resonant excitation of sulfur 2*p* and 2*s* orbitals was probed with photon energies in the ranges 162–175 eV and 214–226 eV, respectively. At both the 2*p* and 2*s* edges, Time-Resolved Near Edge X-ray Absorption Fine Structure spectra (TR-NEXAFS) were collected by scanning time delay and X-ray photon energy. Figure 1 shows a sketch of the probing scheme along with the relevant molecular orbitals. Together with the electron kinetic energy, we measured a three-dimensional data set. To obtain a measure for absorption, we integrate the electron yield over the full kinetic energy range for different photon energies and pump–probe delay settings. In this paper, we only concentrate on these NEXAFS-yield measurements.

**Figure 1:**
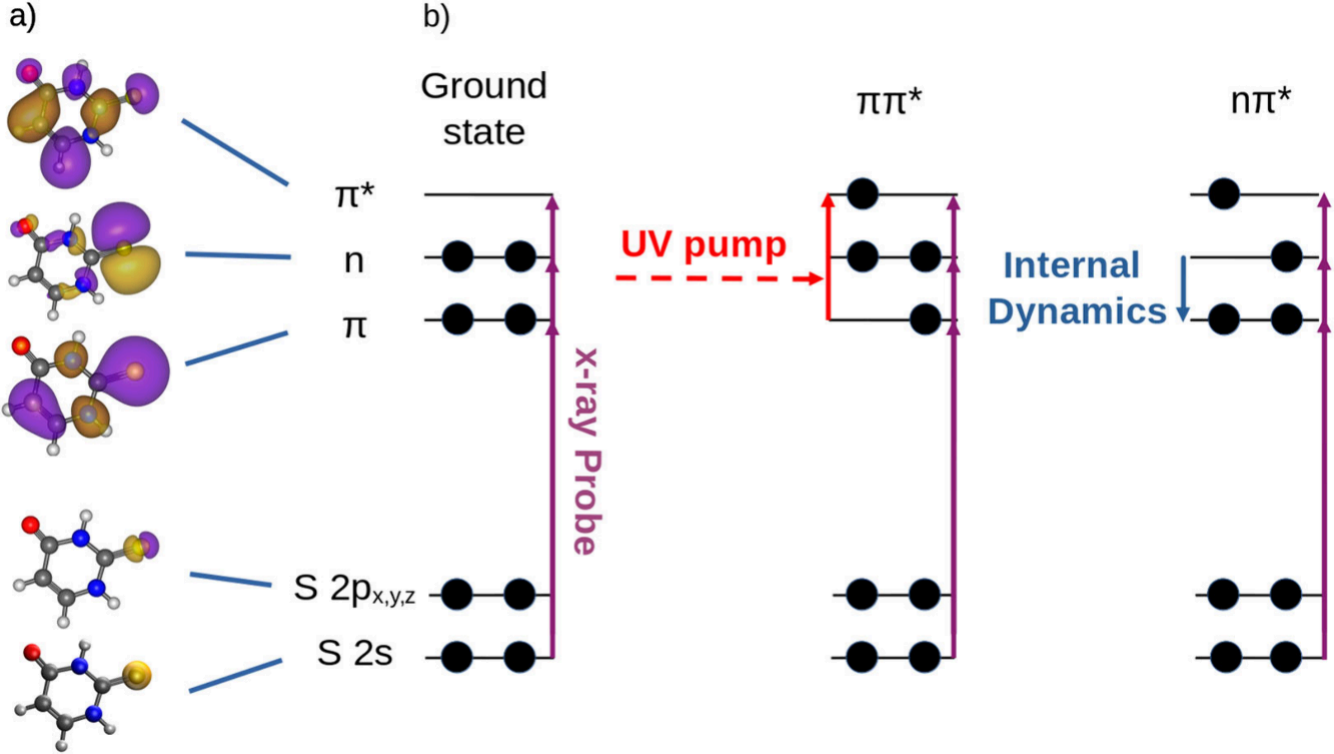
a) Molecular valence orbitals (π, *n*, π*) and core orbitals (Sulfur 2s and 2p) of 2-TU, obtained by a Hartree–Fock calculation using the 6-31++G** basis set. b) Sketch of the experimental scheme. A UV-pump/X-ray probe setup is used. A UV pump pulse is used to initiate the ultrafast dynamics. The X-ray probe pulse resonantly excites the 2-TU molecule to one of the valence orbitals from either the 2p or 2s core orbitals. By change of the X-ray photon energy, multiple orbitals can be probed. Subsequent changes in the valence orbital occupation state can be observed by tracking the X-ray absorption as a function of the pump–probe delay. The X-ray absorption is determined by measuring the total photo- and Auger electron yield in a magnetic bottle electron spectrometer.

To induce ultrafast dynamics, the sample was photoexcited to the ππ* state with a UV pump laser. This laser had a center wavelength of 269 nm and a pulse duration of 80 fs, delivering around 1 μJ of energy for each shot in a 50 μm focus. UV power scans on the time-dependent features were carried out to ensure excitation in the linear regime.

The FLASH2 FEL delivers so-called pulse trains containing 70 X-ray shots per train at a train frequency of 10 Hz, for a total of 700 shots/sec. The X-ray pulse duration has been estimated to be <50 fs, with a shot energy of ∼3.5 μJ, and was linearly polarized along the axis of the MBES. The photon energy is controlled with the use of the FLASH2 variable gap undulators,^34^ allowing for energy-dependent probing. The X-ray probe spot size was chosen to be slightly smaller than that of the UV focus.

To obtain a difference spectrum, the UV laser operated at half the effective repetition rate of the FEL so that every second X-ray shot was unpumped (i.e., X-ray only). The pump–probe spectra are then obtained as the difference between even and odd numbered shots. The data set presented in this work is obtained by combining ∼10^8^ individual X-ray shots, which is on the order of 30 h of data acquisition.

The kinetic energy of the produced photon and Auger electrons was recorded using a MBES (magnetic bottle electron spectrometer). This spectrometer type offers a large collection efficiency so that several tens of electrons can be collected for each FEL shot. To improve the energy resolution of the MBES, a retardation voltage of either −90 or −10 V (for the 2p and 2s regimes, respectively) was applied to the electrons in order to increase their time of flight. The energy resolving power of the MBES (*E/ΔE*) was determined with Kr MNN Auger lines to be 40 at 0 V retardation.^36^

The data was postprocessed to improve time and energy resolution. A Bunch Arrival Monitor (BAM)^38^ instrument was used to measure X-ray/UV delay jitter. The data set was rebinned taking the BAM information into account to improve pump–probe delay resolution. With this method, we arrive at a time resolution of 150 fs of fwhm (extracted from the rise time of the strongest pump–probe feature). The photon energy has been calibrated using data from the OPIS instrument.^39,40^

The bin size was adjusted to ensure that each energy and delay bin contained the same number of X-ray shots. During both delay and photon energy scans, the ordering of the measurement steps was randomized in order to minimize systematic effects.

Error bars have been estimated using the bootstrap method,^41^ with each error bar representing the standard deviation of 70 bootstrap samples.

In order to calibrate the temporal overlap between the UV and X-ray pulses, time zero measurements were conducted independently from the NEXAFS scan. In these scans, the photon energy has been set above the 2*p*/2*s* edges, and the nonresonant sulfur 2*p* Auger spectrum has been measured. The time-dependent dynamics of this feature has been reported in ref (26). The zero-delay position was determined by maximizing the cross-correlation between a step function and the appearance of an energy shift (not a change in the absolute signal) in the Auger electron spectrum. Since the 2*p* Auger XPS feature is present in both photon energy regimes that were scanned, this method was used throughout the experiment to determine time zero in both photon energy regimes. This consistent timing method, independent of the NEXAFS data, allows for a direct comparison between the two data sets. Examples of the results from our time-zero estimation procedure can be found in the Supporting Information.

Due to the constraints imposed on the data collection from the limited amount of beamtime available, we decided to concentrate on relatively narrow photon energy ranges to optimize for signal-to-noise. The 2*p* edge resonances (214–226 eV) are the strongest absorption features observed and have been used as a marker of the ground state depletion. The pre-edge region of the 2*s* edge (162–175 eV) was used as a probe for the nπ* state, given the high wave function overlap between the core 2*s* orbital and the lone pair *n* orbital visible in this spectral region.

For reference, we also collected high-resolution NEXAFS spectra using the hemispherical photoelectron spectrometer at the PLEIADES beamline of the SOLEIL synchrotron for electron yield measurements.^42^ The spectra were recorded with an energy resolution of 0.25 eV.

The element and site sensitivity typical for X-ray spectroscopy allow us to investigate the molecular dynamics in the vicinity of the sulfur atom. The sulfur X-ray photoelectron spectrum for 2-thiouracil has been reported in previous works,^43^ and its time-dependent features have been studied in the nonresonant case.^25,26^ We investigate the time dependence of X-ray absorption by integrating the photoelectron yield.

The results from the investigation at the sulfur 2*p* edge are shown in Figure 2. We subtracted the “UV off” (no UV pump pulse) spectrum from the “UV on” data, resulting in a difference spectrum. The UV pump pulse precedes the X-ray probe for positive delays. This difference spectrum is equal to *f(ES-GS)*, where *ES* and *GS* are the excited state and ground state spectra, respectively, and *f* is the fraction of molecules that are excited by the UV pump pulse, estimated to be about 0.13.^26^

**Figure 2:**
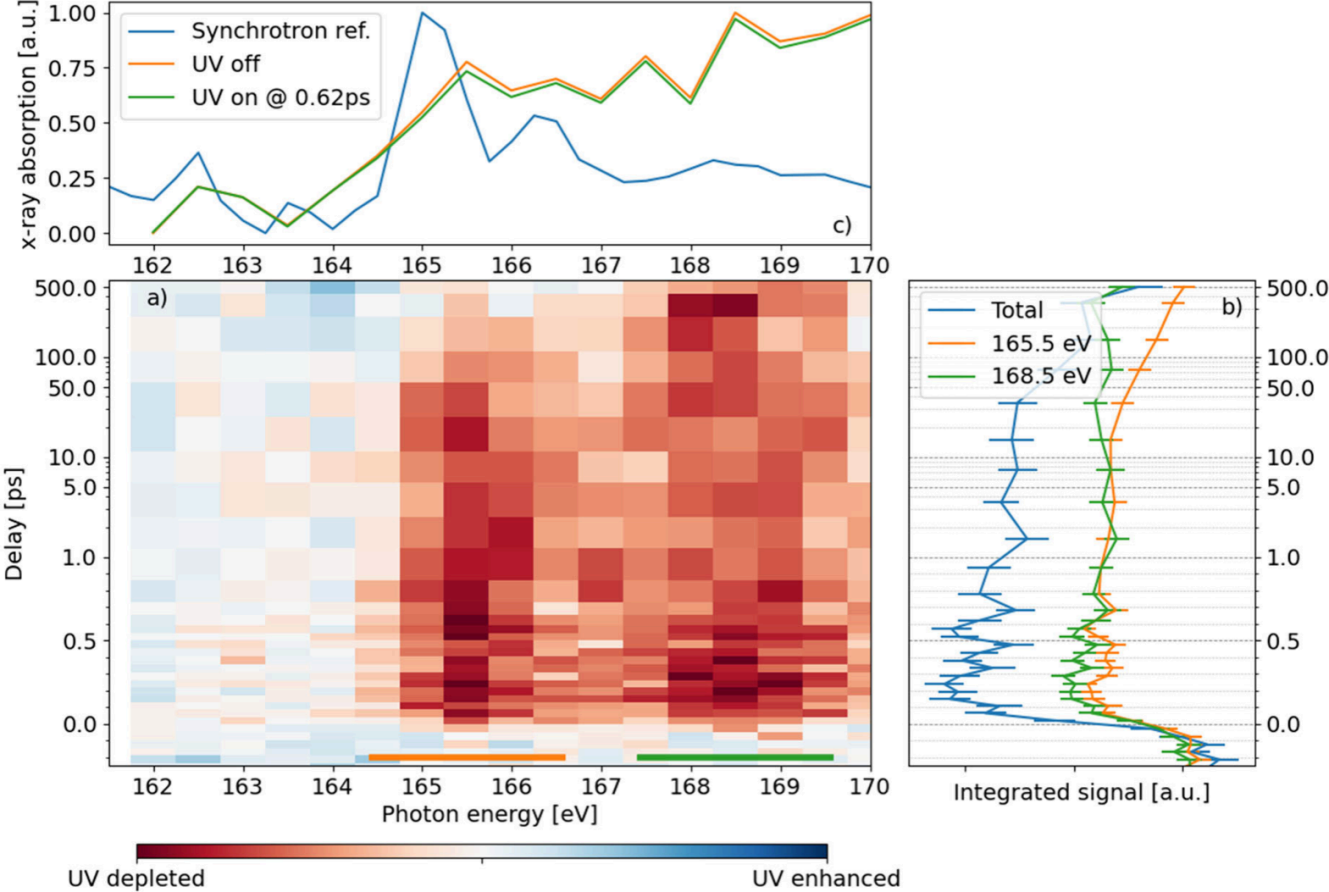
a) Time resolved difference NEXAFS spectrum of 2-thiouracil at the sulfur 2*p* edge. The spectrum is obtained by integrating the differential intensity in the total electron yield while scanning over pump–probe delay and X-ray photon energy. Two main features are visible, centered at 165.5 and 168.5 eV, respectively, where we observe a UV-induced depletion of the photoelectron signal. Both regions have an onset within the first 100 fs, with the 165.5 eV region decaying on a 100 ps scale and the 168.5 eV feature persisting for all delays scanned. b) Integral of the differential signal for different photon energy regions, indicated by the colored bar in a), vs delay. Oscillations in the overall intensity are visible (blue line) with a 200–250 fs period. The comparison between the 165.5 and 168.5 eV regions (green and orange, respectively) shows the difference in the decay rate. c) Comparison of our FEL data with the synchrotron reference spectrum from SOLEIL, showing more sharply the position of the pre-edge resonances. Although the energy resolution of the FEL data does not resolve the features, a general increase in absorption can be seen over the 2*p* edge.

Figure 2a shows a false-color map of the electron yield, integrated over all kinetic energies, vs pump–probe delay and photon energy. We observe a UV-induced depletion of the difference signal in red on the false color map, concentrated in two bands, centered at photon energies of 165.5 and 168.5 eV, respectively. Both regions show an ultrafast rise on a sub-100-fs time scale, but they have different decay behavior. The 165.5 eV band decays on a 100 ps time scale, while the 168.5 eV feature persists for all time delay scanned - up to 500 ps - as evidenced by the integral plots (green and orange lines in Figure 2b). Moreover, we observe oscillations in the overall bleach intensity on an ∼200 fs time scale that are best visible when integrating over the entire photon energy range (162–170 eV, blue line in Figure 2b). Figure 2c shows the absorption spectra with and without UV excitation (green and orange curves, respectively) as compared with the absorption spectrum measured at the synchrotron (blue). Despite the X-ray bandwidth around 2 eV, we can still discern the doublet structure split by 1.2 eV. The latter is associated with the sulfur 2*p* spin–orbit splitting.^44^

We now focus on the 2*s* edge, where we concentrate on the pre-edge region to find a sulfur 2*s* core to *n* orbital transition, motivated by our previous studies on thymine.^31^ Analogous to the 2*p* edge, a delay vs photon energy map has been constructed from the difference photoelectron spectrum, which is shown in Figure 3a). The most prominent feature is the appearance of a pump-induced increase in the X-ray absorption in the range of 222 to 224 eV, rendered with blue color in the difference map. The feature onset is delayed by about 150 fs with respect to time zero and persists for all the delay points available, up to 100 ps (the zero pump–probe delay point is set with the same procedure for both data sets, as discussed in the previous section). The amplitude of this feature shows modulations in the intensity profile with a period of ca. 200 fs, that are visible in Figure 3b. The comparison of the UV on and UV off absorption spectra from FLASH (green and orange line, respectively) with the synchrotron data shows that the time-dependent signals are lower in energy than the pre-edge region of the ground state NEXAFS spectrum. The time-dependent features are separated roughly by a UV photon energy of 4.5 eV from the ground state NEXAFS maxima.

**Figure 3:**
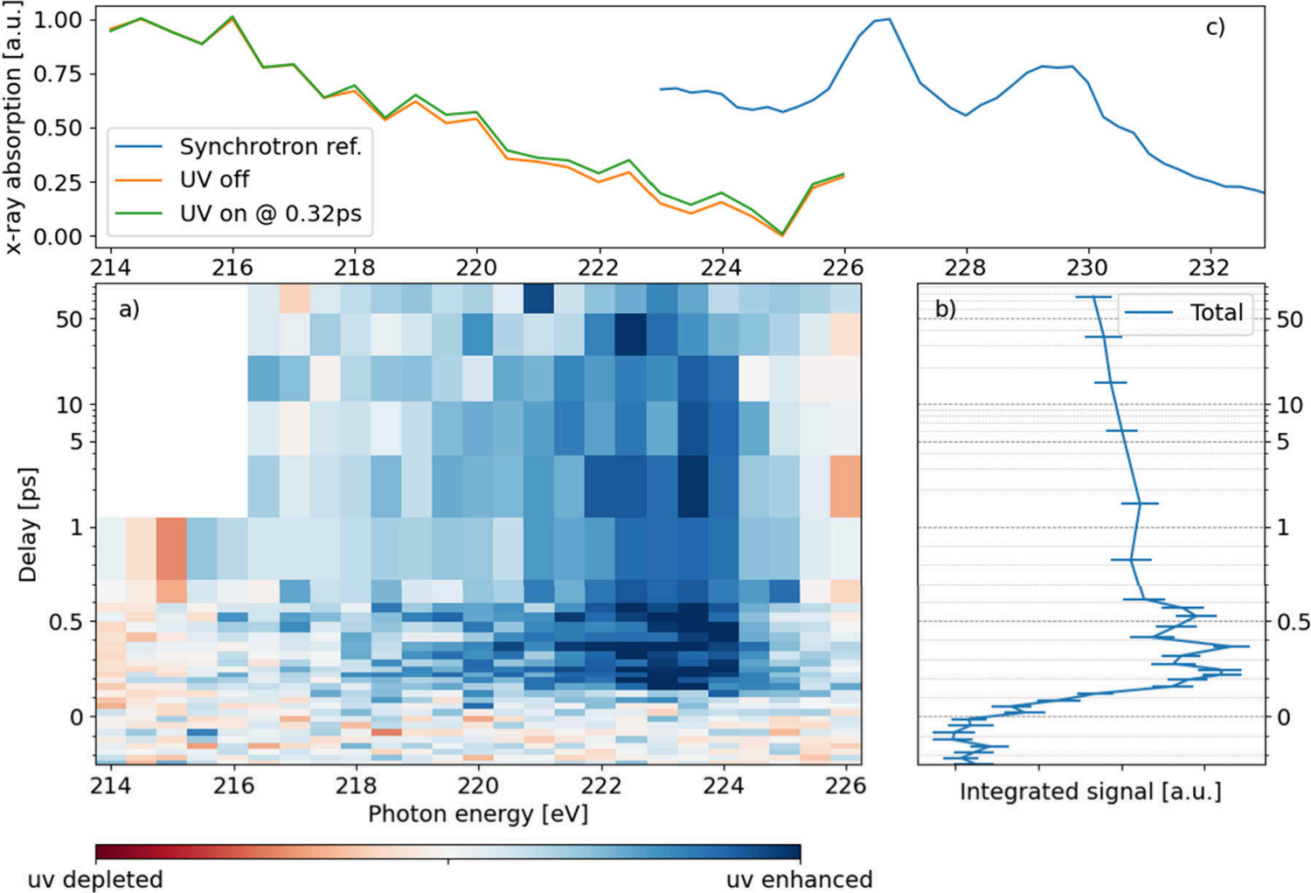
a) Time resolved difference NEXAFS spectrum of 2-thiouracil at the sulfur 2*s* edge. The spectrum is obtained by integrating the differential intensity in the total electron yield, including the 2*p* photoelectron lines, while scanning over pump–probe delay and X-ray photon energy. The feature appearing at 223 eV indicates an UV-induced increase in X-ray absorption, beginning at 150 fs and persisting at long delays, up to 100 ps. b) Integral of the differential signal for photon energies in the range 217–226 eV. For short time delays, an oscillation in the signal intensity is visible, with a period of about 200 fs. c) Comparison of our FEL data with the synchrotron reference spectrum from SOLEIL. The observed time-dependent feature appears at lower photon energies than the ground state absorption resonances present in the reference spectrum.

We compare the spectrally integrated signals as a function of the delay at the two edges in Figure 4. The bleach in the S *2p* (L2/3) NEXAFS spectrum (blue line) is shown with an inverted sign. As remarked above, its onset is associated with the time-overlap of the UV pump and X-ray probe pulse. The time-delayed increase in the S *2s* (L1) NEXAFS spectrum (orange line) is scaled such that the two signals have the same average level after the initial rise. The delay between the two rise times was determined to be 102 ± 11 fs by an error function (ERF) fit of the two data sets.

**Figure 4:**
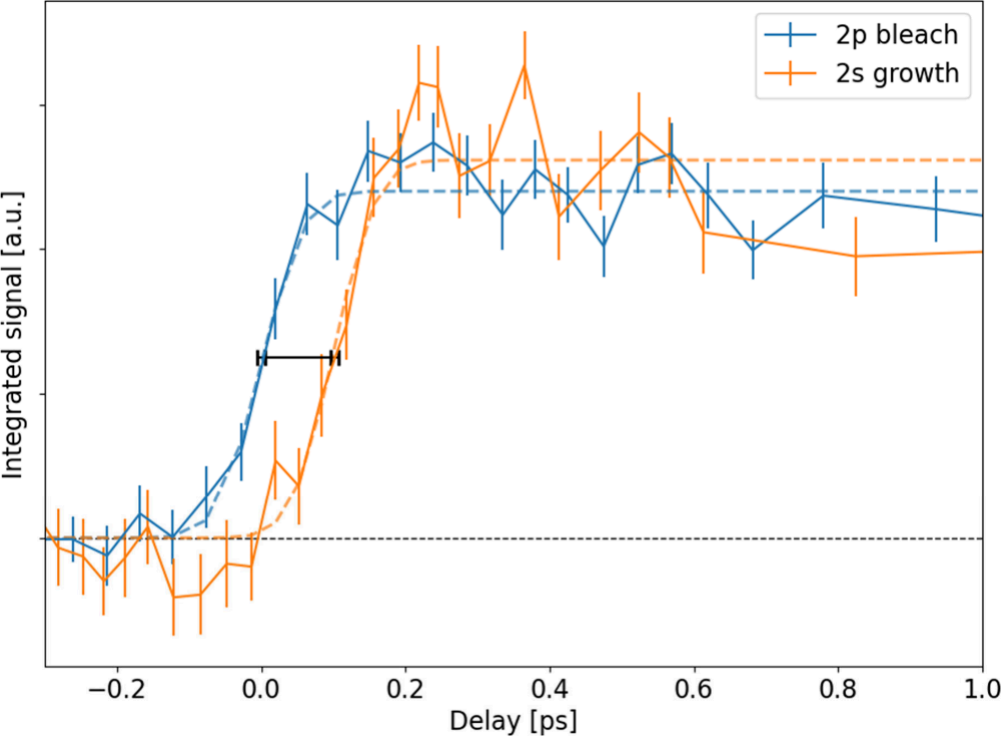
Comparison of the appearance time of the two TR-NEXAFS features shown in Figures 2b and 3b. After the immediate rise of the ground state bleach from the 2*p* NEXAFS (blue line), there is a delayed growth in the 2s NEXAFS (orange line). The dotted lines indicate an ERF fit of the observed data, and the black bar indicates the estimated rise time delay. Moreover, for time delays in the range 0.2–0.6 ps, oscillations in the amplitude of both signals can be identified.

We will now discuss the measured results in the context of the proposed dynamics for the molecule. The measured features in the TR-NEXAFS scans, at both sulfur edges, indicate ultrafast changes in the valence electronic structure. In a single-electron orbital picture, UV-induced valence excitation changes the available orbitals for the resonant core to a valence transition probed by the X-rays. Therefore, we use the observed changes in the X-ray absorption yield to study the dynamics in the electronic state of the molecule. A similar approach has already proven successful in the study of the ultrafast dynamics of thymine.^31^

The strongest feature in our data set, the bleach in the sulfur 2*p* NEXAFS spectrum shown in Figure 2, can be used as an indication of the ground state depletion that follows UV excitation. Those features are excitations from the *2p* orbital to Rydberg states (see Table 3 in ref (40)). The modulation of these features to a UV photoexcitation, which is mostly confined to π-π* transitions, exposes the multielectron character of the X-ray absorption transitions. The rise time of this feature is within the time resolution of our measurement and simultaneous to the arrival of the pump pulse and is therefore compatible with the immediate population of the optically active S2 ππ* state. In a single electron picture, the UV-induced decrease in absorption is explained by a partial occupation of the π* orbital. This leads to a reduced signal in the S 2p π* absorption channels.

The S 2*s* NEXAFS spectrum is ideally suited to probe the S1 state, which is dominantly of nπ* character. The *n*-orbital is a lone-pair sulfur orbital that is thus strongly localized at the sulfur atom. The molecular *n*-orbital is resembling a sulfur 3*p* orbital (see Figure 1). In the nπ* state, the lone-pair *n*-orbital is half filled, thus providing an opportunity for allowed absorption from the core sulfur 2*s* orbital into the *n*-orbital. This absorption is particularly strong because of the almost exclusive atomic nature of the core and valence orbitals involved in this transition. These arguments have been implied in the past for explanation of gas-phase and liquid phase absorption spectra^31,45^ and are successful again in the current context.

To get further insight into the spectral features between 221 and 226 eV, we performed equation-of-motion coupled-cluster (EOM-CCSD) simulations of the pump–probe signal for different initial states and geometries for the X-ray probe step. The computational details of these simulations are given in the Supporting Information, and the computed pump–probe pre-edge spectra are shown in Figure 5. Following the excited state geometry optimizations carried out in ref (25), we consider fully optimized structures in the S_n_ and T_n_ potential energy surfaces, denoted S_n,min_ and T_n,min_, as well as local planar minima S_n,pla_ and T_n,pla_. Indeed, since the S_0_ minimum is planar, planar molecular configurations are expected to be most relevant in the short time scale (<100 fs), as confirmed by nonadiabatic surface-hopping simulations at different levels of theory.^29,30^

**Figure 5:**
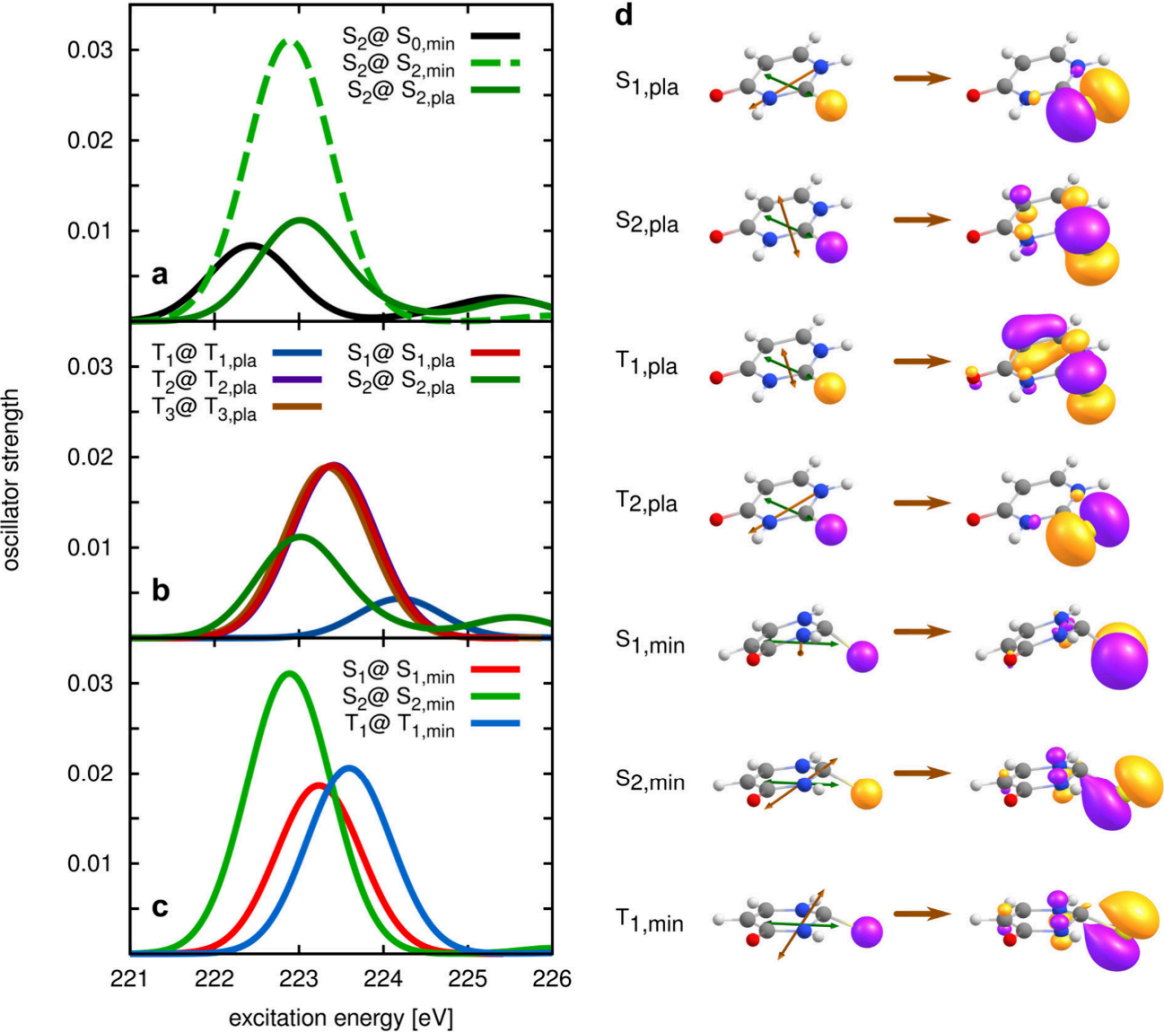
Calculated pump–probe bands at the sulfur 2s edge for different initial valence excited states and different molecular geometries. (a) Spectra for the core transition starting from the S_2_ (ππ*) state at the Franck–Condon point, the planar and nonplanar S_2_ minima; (b) spectra for various single and triplet initial states at the respective planar stationary points; (c) spectra for the initial states S_1_, S_2_ and T_1_ at their respective fully optimized minima; (d) dominant natural transition orbitals associated with the core excitations out of various valence excited states at their respective planar or fully optimized minima. The green arrow depicts the direction of the dipole **μ**_02_, associated with the S_0_ → S_2_ transition. The brown arrows show the transition dipoles for the core–valence excitations, magnified by a factor of 40.

In Figure 5, panel (a) shows the calculated pump–probe spectra for transitions out of the S_2_ state at three different geometries (the Franck–Condon point and the planar and nonplanar minimum), panel (b) depicts the spectra for transitions initiated from the planar minima of the lowest singlet and triplet states, and panel (c) shows the same for the optimized nonplanar structures. The computations predict a rather small increase of intensity for the signal associated with the motion of the molecule from the Franck–Condon zone (black) toward S_2,pla_ (dark green). The intensity is found to increase more significantly, by a factor of approximately 2, during the internal conversion from the S_2_ (ππ*) state to the S_1_ (nπ*) state. This indicates that the delay in the signal at the 2*s* edge relative to the initial excitation is mainly caused by the time required to populate the S_1_ state.

Therefore, we attribute the observed delay of 102 ± 11 fs in the signal rise time of the pre-edge *2s* (L1) NEXAFS increase with respect to time-zero (Figure 4) to the S2 to S1 transition. The time scales predicted by surface-hopping calculations are rather sensitive on the level of electronic structure theory, ranging from 59 ± 12 fs in the case of MS-CASPT2^29^ to 250 ± 65 fs for ADC2 calculations.^30^ Our result, while being compatible with both of them, seems to indicate an intermediate value between the two methods.

An additional contribution to the delayed rise of the signal at the 2*s* (L_1_) edge could be attributed to the signal increase due to the molecular wavepackets passage to a nonplanar minimum (S_2,min_). At this geometry, the signal is stronger than in the S_2,pla_ and also than in any geometry in the S_1_ state. We exclude, however, that this is the dominating reason for the delay. If this channel were the main one, we would observe first a strong rise and then a decay of the spectroscopic signal in the experiment, because the S_2_ state unavoidably decays to lower lying states. In contrast, after the 100 fs buildup, the intensity of the observed signal remains approximately constant. This argument is further strengthened by including the relative alignment of pump- and probe dipole moments, as will be detailed below.

The finite signal due to the S_2_ population at the Franck–Condon point in the simulations suggests a (weak) pre-edge signal in the *2s* (L_1_) NEXAFS spectrum from time-zero before the delayed increase. Considering a planar geometry for the molecule, as is the case in the early stages of the relaxation dynamics, this S_2_ feature is predicted to have an intensity at least 50% lower than that of the lower electronic states, which could be in principle visible as a small initial increase in the *2s* (L_1_) NEXAFS spectrum at zero delay. Given our error bars, such a feature, if present, should have an intensity at least 3 times lower than the main absorption feature.

To investigate further which factors affect the intensity of the absorption from the valence excited states, we analyzed the dominant core excitations in terms of natural transition orbitals (NTOs).^46^ The dominant NTOs, depicted in Figure 5d, can be regarded as the effective orbitals involved in one-electron transitions. The occupied NTO is, of course, the 2*s* orbital of sulfur, whereas the arrival valence orbital is delocalized and depends on the initial valence excited state. The sketches at the left side of Figure 5d also depict the transition dipole moments **μ**_02_ (green), associated with the S_0_ → S_2_ (pump) excitation and the various dipoles **μ**_*nm*_ (brown) associated with the (probe) transitions from valence excited to core excited states. It is worth emphasizing that the intensity of the transitions must be computed as the product between the oscillator strength for the core–valence transition and an orientational factor κ given as κ = [1 + 2 cos^2^α]/3, where α is the angle between the transition dipole moments for the pump and probe steps, **μ**_02_ and **μ**_*nm*_, respectively. This factor accounts for the fact that the pump (probe) pulse excites more favorably the molecules whose orientation is such that **μ**_02_ (**μ**_*nm*_) is parallel to the field and that the pump and the probe fields have parallel polarization in the experiment.

For the probe transitions starting from the S_1_ and T_2_ states (nπ*) the arrival NTO is an n-type orbital well localized at the sulfur atom, at both the planar (S_1,pla_ in dark red and T_2,pla_ in purple) and nonplanar (S_1,min_ in light red) minima of the nπ* states.

For transitions out of ππ* states, the arrival NTO is a π orbital mainly located around the C=S bond, with some degree of delocalization on the ring. At planar geometries (S_2,pla_ in dark green and T_1,pla_ in dark blue) the polarization of the core excitations is exactly perpendicular to that of the S_0_ → S_2_ transition. This yields the minimum possible value for the orientational factor *κ.* In contrast, the transition dipole for the core excitations out of nπ* states lies in the plane of the molecule. Although this dipole is not parallel to **μ**_02_, the fact that they are not orthogonal contributes in making the core transitions from nπ* states more intense.

The higher intensity of the pump–probe spectrum at the nonplanar S_2_ minimum (S_2,min_ in dashed light green) is explained by the fact that this structure involves a strong out-of-plane distortion of the C=S bonds that partially aligns the dipoles associated with the pump and probe transitions. The lower intensity of the core excitation from the planar T_1_ minimum (T_1,pla_ in dark blue) is due to the fact that the arrival π orbital is significantly delocalized over the ring, leading to a decreased overlap with the core 2*s* orbital.

Finally, we discuss the oscillations of photon-energy integrated absorption signals observed in both the 2*p* (L2,3) and 2*s* (L1) regions, which appear to be of a similar period (see Figure 4). The signal in the 2*s* region shows a higher modulation amplitude, with similar uncertainty bars as for the 2*p* region. Oscillations begin at a pump–probe delay of ∼200 fs, exhibit a 200 fs period, and continue at least up to 600 fs; at this delay the temporal density of our data set decreases. Oscillations of the same behavior have been previously observed by us in the shift of the S 2*p* X-ray photoelectron spectrum (XPS) after UV excitation, where we found a strong dependency of the XPS shift on electronic state and little dependency on geometry variation.^25^ Therefore, it is unlikely that the oscillations are due to the ground state wave packet being perturbed by the pump pulse. Indeed, the comparison with dynamical simulations available from the literature and our own electronic structure calculations allowed us to associate the oscillations with the population in the S_1_nπ* state.^29,30^ With the present results, this attribution is further supported by the delayed start of the oscillation in the 2*p* (L2,3) NEXAFS (blue line), where the initial crest extends to earlier delays, indicating that the oscillation starts after the ππ* to nπ* transition.

The calculations in refs (29 and 30) do not indicate that one particular state acts as a counterpart to the S_1_ oscillation. Based on the theoretical results shown in Figure 5(b) and 5(c), the most significant difference in intensity relative to the S1 state signal occurs at the planar minimum of the T_1_ surface. This suggests that the observed oscillations are most likely due to a coherent population exchange between the S_1_(nπ*) and T_1_(ππ*) states, consistent with the El-Sayed rule. This interpretation holds if a large fraction of molecules maintains a planar geometry (where the transition intensity out of T_1_ is weak) for at least 600–700 fs. This mechanistic behavior aligns well with the structural findings of the nonadiabatic surface-hopping simulations. Furthermore, the validity of the El-Sayed rule for 2-TU is confirmed by the calculated spin–orbit couplings, reported in the Supporting Information.

An alternative explanation for the observed oscillations, given that they are present in both signals (π* bleach and *n* growth), is a coherent population exchange with the ground state S_0_, which is the only state in which we do not expect any differential absorption signal in either the 2*s* or 2*p* regions. Our past XPS study showed up to 30% relaxation into the hot ground states.^25^ However, one expects a large vibrational reorganization energy associated with the relaxation to S_0_, which favors vibrational dephasing and makes an oscillatory population exchange unlikely. For the oscillation related to the S_1_ state dynamics, a few coherent oscillations can be followed, as the dephasing is smaller due to considerably less vibrational reorganization energy.

In this work, we studied the photoinduced dynamics of 2-thiouracil by time-resolved NEXAFS. By comparing the rise time in the pump–probe features observed at the sulfur 2*s* and 2*p* edges, we were able to estimate a time constant for the ππ–nπ* transition of 102 ± 11 fs. Furthermore, we observe coherent oscillations in the population of the S_1_ state that are reminiscent of the previously reported oscillations in the time-resolved XPS spectra.^25^

Our previous nonresonant XPS and Auger studies^25,26^ on the same molecule showed that X-ray methods can give localized information on electronic charge density and nuclear motion, respectively. In this work, we used resonant X-ray absorption to investigate the population of electronic states with a significant orbital contribution at the probed sulfur location. The NEXAFS data allow for another point of view in the understanding of such molecular dynamics, providing information on the changes of valence orbital occupation during the photorelaxation. This study demonstrates how combining X-ray spectroscopic investigations targeting different observables can provide both electronic and structural insights into ultrafast photoinduced processes in relevant organic chromophores.

## Supporting Information Available

Computational details, singlet–triplet spin–orbit coupling values, detailed description of experimental time-zero calibration procedure. (PDF)
