# Supplementary material for: Direct Observation of the ππ* to nπ* Transition in 2-Thiouracil via Time-Resolved NEXAFS Spectroscopy
Source: J Phys Chem Lett. 2025 Apr 15;16(16):4038–46. doi: 10.1021/acs.jpclett.5c00544 (PMC12035857; doi:10.1021/acs.jpclett.5c00544)
Supplement: Supplementary file 1 — jz5c00544_si_001.pdf [file jz5c00544_si_001.pdf]

Supporting Information for "Direct observation of $\pi\pi^*$ to $n\pi^*$ transition in 2-thiouracil via time resolved NEXAFS spectroscopy"

Fabiano Lever[1,*], David Picconi[2], Dennis Mayer[1], Skirmantas Ališauskas[1], Francesca Calegari[1,3], Stefan Düsterer[1], Raimund Feifel[4], Marion Kuhlmann[1], Tommaso Mazza[5], Jan Metje[6], Matthew S. Robinson[5,3], Richard J. Squibb[4], Andrea Trabattoni[1,7], Matthew Ware[8], Peter Saalfrank[6], Thomas J. A. Wolf[8] and Markus Gühr[1,*]

[1] Deutsches Elektronen-Synchrotron DESY, Hamburg, 22607, Germany
[2] Heinrich-Heine University, Düsseldorf, 40225, Germany
[3] The Hamburg Centre for Ultrafast Imaging, Hamburg, 20148, Germany
[4] University of Gothenburg, Gothenburg, 405 30, Sweden
[5] European XFEL, Schenefeld, 22869, Germany
[6] University of Potsdam, Potsdam, 14469, Germany
[7] Leibniz University Hannover, Hannover, 30060, Germany
[8] Stanford PULSE Institute, SLAC National Accelerator Laboratory, Stanford, California 94305, USA

** Corresponding authors: fabiano.lever@desy.de, markus.guehr@desy.de*

Computational details for the 2*s* edge absorption calculations

Optimized structures of the ground stateand the different singlet and triplet excited states of 2-thiouracil were obtained in a previous work [1] using (equation-of-motion) coupled-cluster calculations ((EOM)-CCSD) with the 6-31++G** basis set. While the S_0 minimum was found to be planar, the structures of the S_1, S_2 and T_1 minima present significant out-of-plane pyramidalization of the C=S bond.
Since the initial structure is planar, it is anyhow expected that a significant fraction of molecules retains a planar geometry at least for the first hundreds of fs after the $S_0 \rightarrow S_2$ photoexcitation. Therefore, the excited states were also optimized by starting from planar structures to find low energy planar stationary points (called "planar minima" hereafter). These optimized planar structures are denoted $S_{n,pla}$ and $T_{n,pla}$ in the main text, whereas the fully optimized minima are called $S_{n,min}$ and $T_{n,min}$. At all the studied geometries the states S_1 and T_2 have $n\pi^*$ character, while S_2, T_1 and T_3 are associated to $\pi\pi^*$ transitions.

To model the pump-probe spectra at the sulfur 2*s* edge, we considered only pre-edge transitions, which start from the valence excited states and terminate into the core excited states that have the same valence configuration of S_0. The calculations were performed using the package Q-Chem 6.2 [2]. At a given geometry, the valence excited states were calculated with the standard EOM-CCSD theory [3], whereas the core excited states were obtained by invoking the core-valence separation starting from the ground state CCSD wavefunction [4]. Following the recommendations or Ref.[5], the 6-311G basis set was used for the H atoms, and the largest 6-311+G(3df) basis was used for the C, N and O atoms; the basis set adopted

for the S atom was 6-311+G(3df) with the core functions uncontracted.
The state-to-state transition dipole moments for the core excitations were computed using the implementation available in Q-Chem for EOM-CCSD wavefunctions.
The intensity of the transitions was computed as the product between the oscillator strength for the core-valence transition, and an orientational factor κ given as

$$\kappa = \frac{1}{3}\left[1 + 2\cos^2\alpha\right]$$

where α is the angle between the transition dipole moment $\boldsymbol{\mu}_{02}$, associated to the $S_0 \rightarrow S_2$ (pump) excitation, and the various dipoles $\boldsymbol{\mu}_{nm}$ associated to the (probe) transitions from valence excited to core excited states. This factor accounts for the fact that pump (probe) pulse excites more favourably the molecules whose orientation is such that $\boldsymbol{\mu}_{02}$ ($\boldsymbol{\mu}_{nm}$) is parallel to the field, and that the pump and the probe fields have parallel polarization in the experiment.

Spin-orbit couplings between valence excited states

The spin-orbit couplings between S0 and the $n\pi^*$ or $\pi\pi^*$ valence excited states, as well those between the excited states, are calculated using non-relativistic (EOM)-CCSD/6-31++G** wavefunctions, as matrix elements of the Breit-Pauli Hamiltonian, where the two-electron part is evaluated using a mean field approximation [6]
The resulting matrix elements are reported in Table S1, and agree well with the results obtained using multi-state complete active space perturbation theory (MS-CASPT2) or the algebraic diagrammatic construction to second order (ADC(2)) [7]. The results confirm the validity of El-Sayed rules for 2-thiouracil, that predicts that the intersystem crossing occurs most likely between states of different character ($n\pi^*$ and $\pi\pi^*$).

Table S1. Spin-orbit coupling terms (cm^{-1}) between the lowest singlet and triplet valence excited states computed at different molecular geometries.

Geometry	$S_0 - {}^3n\pi^*$	$S_0 - {}^3\pi\pi^*$	${}^1n\pi^* - {}^3n\pi^*$	${}^1n\pi^* - {}^3\pi\pi^*$	${}^1\pi\pi^* - {}^3n\pi^*$	${}^1\pi\pi^* - {}^3\pi\pi^*$
$S_{0,pla}$	111	0	0	133	125	0
$S_{1,pla}$	113	0	0	150	135	0
$S_{2,pla}$	101	0	0	144	140	0
$T_{1,pla}$	110	0	0	122	108	0
$T_{2,pla}$	114	0	0	147	134	0
$S_{1,min}$	144	91	43	147	127	46
$S_{2,min}$	168	143	10	161	152	30
$T_{1,min}$	133	70	13	155	139	25

Comparison between pump-probe spectra at the sulfur 2s edge calculated using equation-of-motion coupled-cluster or time-dependent density functional theory

In this section we compare the pump-probe NEXAFS spectra at the S 2s edge calculated using EOM-CCSD with those obtained by an approach based on time-dependent density functional theory (TDDFT) at the same geometries.
To this end, following the approach of Besley et al. [8] the Tamm-Dancoff approximation was used in conjunction with the short-range corrected functional SRC1-R1, and a truncated excitation space to deploy core-valence separation and target exclusively the one-electron

excitations out of the 2*s* core orbital.

For the transitions starting from valence excited states, the reference Kohn-Sham wavefunction was found by unrestricted “Δ-SCF” calculations imposing orbital occupancies which mimic those of the excited states. To this end, the maximum-overlap-method was used [9]. The stick spectra obtained by TDDFT were shifted by 4 eV and convoluted with a Gaussian with a standard deviation of 1.7 eV to match the experimental linewidth. The pump-probe spectra states were btained by difference between the excited state and the ground state spectral profiles.

The TDDFT electronic structure calculations employed the 6-311++G** basis set, and were performed using the Q-Chem package version 5.4.

The pump-probe spectra computed at the EOM-CCSD and the TDDFT level are compared in Fig. S1. Despite minor shifts in the positions of the bands, the intensity pattern is similar for the two types of calculation. This reinforces the interpretation of the experimental results given in the main manuscript. In particular, although being based on spin-contaminated valence excited states, the TDDFT simulations replicate the intensity increase upon the $S_2 \rightarrow S_1$ internal conversion obtained by the coupled-cluster theory.

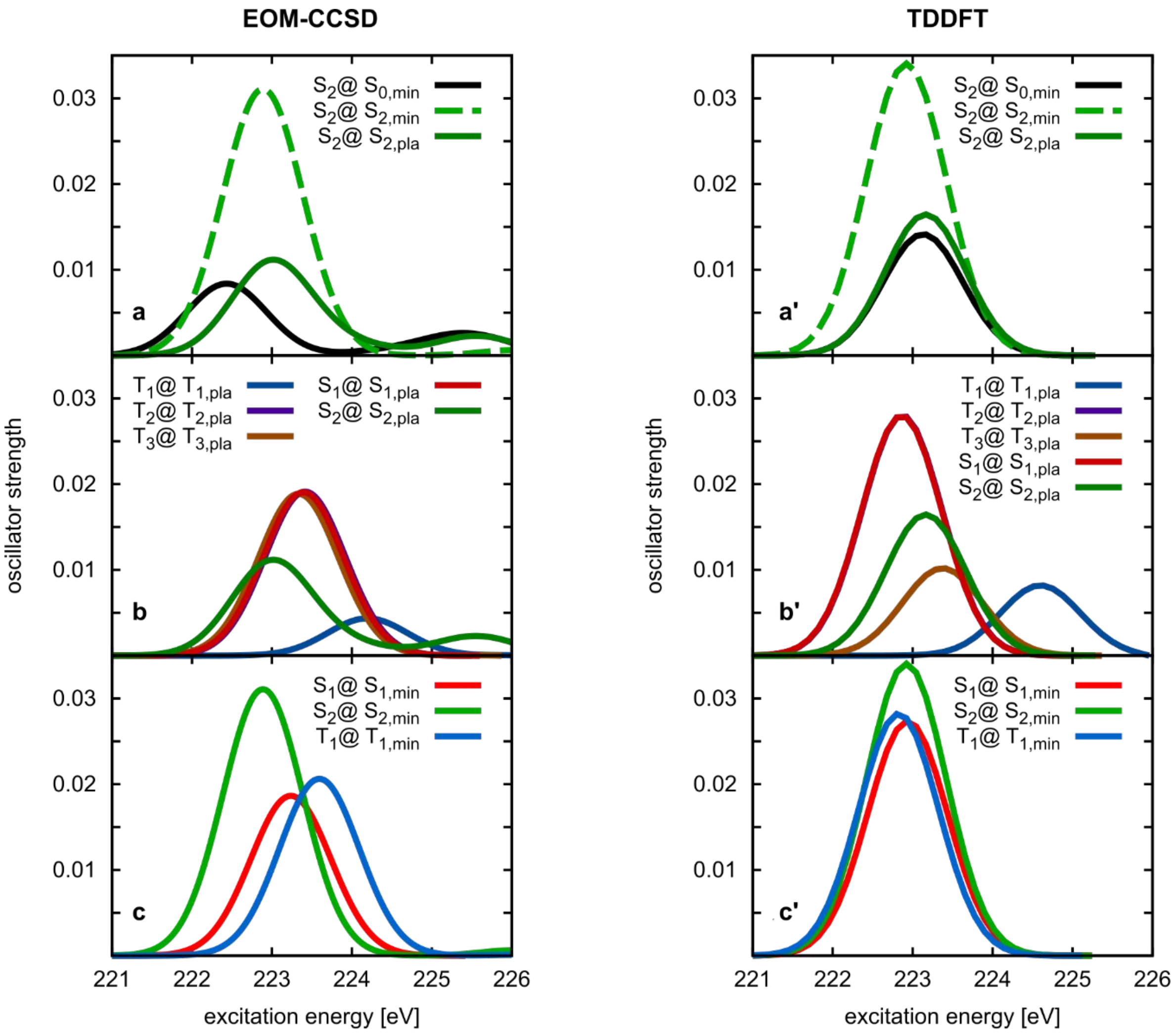

Fig. S1. Calculated pump-probe bands at the sulfur 2s edge for different initial valence excited states and different molecular geometries. The spectra are computed at the EOM-CCSD (a-c) or TDDFT (a’-c’) level.

Details of the time-zero estimation procedure

In order to calibrate the time axis of both datasets (2s and 2p) to the same timing reference, the same procedure has been used to determine a time-zero reference in both regimes.

We base our timing calibration on the appearance of a UV-induced shift in the 2p Auger feature, as this feature can be observed in both energy regimes by setting the photon energy to be above the 2p absorption edge. Since the photon energy is controlled by adjusting the undulator gap in the FEL, we do not expect such operation to have aany effect on the timing.

After the initial estimation of our time-zero delay position, additional time-zero scans were performed periodically to ensure that timing drifts that are not picked up by our diagnostic tools (such as the Bunch Arrival Monitor [10]), could be detected. In these measurements, the photon energy remains fixed above the 2p bining energy, and pump-probe delay was scanned over a small region around the estimated time-zero position. The maximum available UV pump power was used, to ensure a good contrast in the differential signal.

The photoelectron spectrum is recorded in our MBES (see the methods sections for more information on the setup), and the Auger feature is isolated based on the kinetic energy of the observed electrons.

Figure S2 shows two examples of such time-zero runs for the two regimes. The blue line shows the position of the time zero line, estimated by an automatic routine that requires no additional human input. This estimation is obtained by maximizing the cross correlation of a step function with the differential signal intensity (i.e. the integral over the kinetic energy axis of the absolute value of the difference signal).

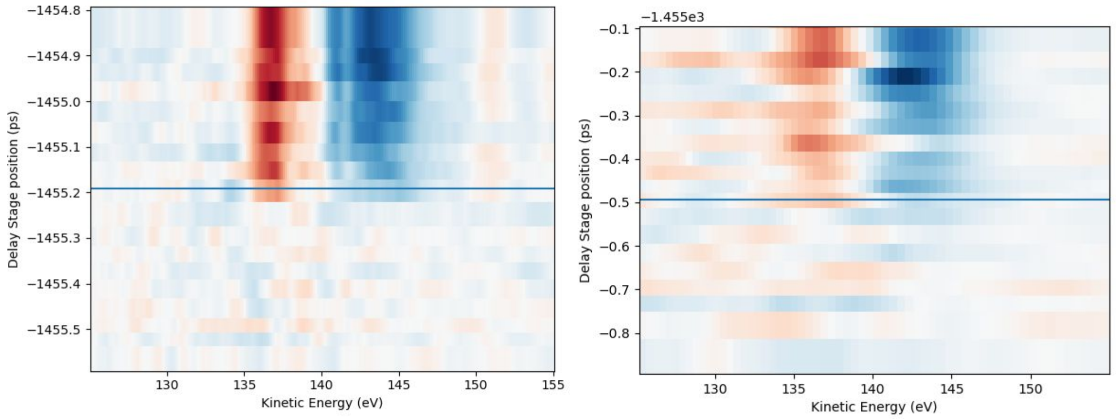

Fig S2. Example results from the time-zero estimation procedure, in the two photon energy regimes at the 2p (left) and 2s (right) absorption edges. The blue line correspond to the estimated time zero position, calculated automatically by a procedure that requires no human input.

We repeaded time-zero scan before each major data acquisition run, with at most a few hours between subsequent time-zero checks. This allows us to observe and correct any long term timing drifts. All individual measurement runs that we used to construct the datasets presented in the manuscript have been referenced to their corresponding time-zero scan.

Bibliography

1. Mayer, D., Lever, F., Picconi, D., Metje, J., Alisauskas, S., Calegari, F., Düsterer, S., Ehlert, C., Feifel, R., Niebuhr, M., Manschwetus, B., Kuhlmann, M., Mazza, T., Robinson, M.S., Squibb, R.J., Trabattoni, A., Wallner, M., Saalfrank, P., Wolf, T.J.A., and Gühr, M. (2022) Following excited-state chemical shifts in molecular ultrafast x-ray photoelectron spectroscopy. *Nat. Commun.*, **13** (1), 198.
2. Epifanovsky, E., Gilbert, A.T.B., Feng, X., Lee, J., Mao, Y., Mardirossian, N., Pokhilko, P., White, A.F., Coons, M.P., Dempwolff, A.L., Gan, Z., Hait, D., Horn, P.R., Jacobson, L.D., Kaliman, I., Kussmann, J., Lange, A.W., Lao, K.U., Levine, D.S., Liu, J., McKenzie, S.C., Morrison, A.F., Nanda, K.D., Plasser, F., Rehn, D.R., Vidal, M.L., You, Z.-Q., Zhu, Y., Alam, B., Albrecht, B.J., Aldossary, A., Alguire, E., Andersen, J.H., Athavale, V., Barton, D., Begam, K., Behn, A., Bellonzi, N., Bernard, Y.A., Berquist, E.J., Burton, H.G.A., Carreras, A., Carter-Fenk, K., Chakraborty, R., Chien, A.D., Closser, K.D., Cofer-Shabica, V., Dasgupta, S., De Wergifosse, M., Deng, J., Diedenhofen, M., Do, H., Ehlert, S., Fang, P.-T., Fatehi, S., Feng, Q., Friedhoff, T., Gayvert, J., Ge, Q., Gidofalvi, G., Goldey, M., Gomes, J., González-Espinoza, C.E., Gulania, S., Gunina, A.O., Hanson-Heine, M.W.D., Harbach, P.H.P., Hauser, A., Herbst, M.F., Hernández Vera, M., Hodecker, M., Holden, Z.C., Houck, S., Huang, X., Hui, K., Huynh, B.C., Ivanov, M., Jász, Á., Ji, H., Jiang, H., Kaduk, B., Kähler, S., Khistyaev, K., Kim, J., Kis, G., Klunzinger, P., Koczor-Benda, Z., Koh, J.H., Kosenkov, D., Koulias, L., Kowalczyk, T., Krauter, C.M., Kue, K., Kunitsa, A., Kus, T., Ladjánszki, I., Landau, A., Lawler, K.V., Lefrancois, D., Lehtola, S., Li, R.R., Li, Y.-P., Liang, J., Liebenthal, M., Lin, H.-H., Lin, Y.-S., Liu, F., Liu, K.-Y., Loipersberger, M., Luenser, A., Manjanath, A., Manohar, P., Mansoor, E., Manzer, S.F., Mao, S.-P., Marenich, A.V., Markovich, T., Mason, S., Maurer, S.A., McLaughlin, P.F., Menger, M.F.S.J., Mewes, J.-M., Mewes, S.A., Morgante, P., Mullinax, J.W., Oosterbaan, K.J., Paran, G., Paul, A.C., Paul, S.K., Pavošević, F., Pei, Z., Prager, S., Proynov, E.I., Rák, Á., Ramos-Cordoba, E., Rana, B., Rask, A.E., Rettig, A., Richard, R.M., Rob, F., Rossomme, E., Scheele, T., Scheurer, M., Schneider, M., Sergueev, N., Sharada, S.M., Skomorowski, W., Small, D.W., Stein, C.J., Su, Y.-C., Sundstrom, E.J., Tao, Z., Thirman, J., Tornai, G.J., Tsuchimochi, T., Tubman, N.M., Veccham, S.P., Vydrov, O., Wenzel, J., Witte, J., Yamada, A., Yao, K., Yeganeh, S., Yost, S.R., Zech, A., Zhang, I.Y., Zhang, X., Zhang, Y., Zuev, D., Aspuru-Guzik, A., Bell, A.T., Besley, N.A., Bravaya, K.B., Brooks, B.R., Casanova, D., Chai, J.-D., Coriani, S., Cramer, C.J., Cserey, G., DePrince, A.E., DiStasio, R.A., Dreuw, A., Dunietz, B.D., Furlani, T.R., Goddard, W.A., Hammes-Schiffer, S., Head-Gordon, T., Hehre, W.J., Hsu, C.-P., Jagau, T.-C., Jung, Y., Klamt, A., Kong, J., Lambrecht, D.S., Liang, W., Mayhall, N.J., McCurdy, C.W., Neaton, J.B., Ochsenfeld, C., Parkhill, J.A., Peverati, R., Rassolov, V.A., Shao, Y., Slipchenko, L.V., Stauch, T., Steele, R.P., Subotnik, J.E., Thom, A.J.W., Tkatchenko, A., Truhlar, D.G., Van Voorhis, T., Wesolowski, T.A., Whaley, K.B., Woodcock, H.L., Zimmerman, P.M., Faraji, S., Gill, P.M.W., Head-Gordon, M., Herbert, J.M., and Krylov, A.I. (2021) Software for the frontiers of quantum chemistry: An overview of developments in the Q-Chem 5 package. *J. Chem. Phys.*, **155** (8), 084801.
3. Stanton, J.F., and Bartlett, R.J. (1993) The equation of motion coupled-cluster method. A systematic biorthogonal approach to molecular excitation energies, transition probabilities, and excited state properties. *J. Chem. Phys.*, **98** (9), 7029–7039.
4. Vidal, M.L., Feng, X., Epifanovsky, E., Krylov, A.I., and Coriani, S. (2019) New and Efficient Equation-of-Motion Coupled-Cluster Framework for Core-Excited and Core-Ionized States. *J. Chem. Theory Comput.*, **15** (5), 3117–3133.
5. Sarangi, R., Vidal, M.L., Coriani, S., and Krylov, A.I. (2020) On the basis set selection for calculations of core-level states: different strategies to balance cost and accuracy. *Mol. Phys.*, **118** (19–20), e1769872.
6. Pokhilko, P., Epifanovsky, E., and Krylov, A.I. (2019) General framework for calculating spin–orbit couplings using spinless one-particle density matrices: Theory and application to the equation-of-motion coupled-cluster wave functions. *J. Chem. Phys.*, **151** (3), 034106.
7. Mai, S., Plasser, F., Pabst, M., Neese, F., Köhn, A., and González, L. (2017) Surface

hopping dynamics including intersystem crossing using the algebraic diagrammatic construction method. *J. Chem. Phys.*, **147** (18), 184109.

8. Besley, N.A., Peach, M.J.G., and Tozer, D.J. (2009) Time-dependent density functional theory calculations of near-edge X-ray absorption fine structure with short-range corrected functionals. *Phys. Chem. Chem. Phys.*, **11** (44), 10350.
9. Gilbert, A.T.B., Besley, N.A., and Gill, P.M.W. (2008) Self-Consistent Field Calculations of Excited States Using the Maximum Overlap Method (MOM). *J. Phys. Chem. A*, **112** (50), 13164–13171.
10. Loehl, F., Arsov, V., Felber, M., Hacker, K., Jalmuzna, W., Lorbeer, B., Ludwig, F., Matthiesen, K.-H., Schlarb, H., Schmidt, B., Schmueser, P., Schulz, S., Szewinski, J., Winter, A., and Zemella, J. (2010) Electron Bunch Timing with Femtosecond Precision in a Superconducting Free-Electron Laser. *Phys. Rev. Lett.*, **104** (14), 144801.
